# Thermal performance curves, activity and survival in a free‐ranging ectotherm

**DOI:** 10.1111/1365-2656.70091

**Published:** 2025-07-16

**Authors:** Kristoffer H. Wild, John H. Roe, Jonathan Curran, Phillip R. Pearson, Lisa Schwanz, Arthur Georges, Stephen D. Sarre

**Affiliations:** ^1^ Centre for Conservation Ecology and Genomics, Institute for Applied Ecology University of Canberra Canberra Australian Capital Territory Australia; ^2^ School of BioSciences The University of Melbourne Parkville Victoria Australia; ^3^ Department of Biology University of North Carolina Pembroke Pembroke North Carolina USA; ^4^ Evolution and Ecology Research Centre, School of Biological, Earth and Environmental Sciences University of New South Wales Sydney New South Wales Australia

**Keywords:** accelerometry, behavioural thermoregulation, ectotherm, lizard, movement, performance, radiotelemetry, survival

## Abstract

1. Temperature profoundly influences the distribution and diversity of ectotherms, yet in natural settings, interactions between environmental temperatures, behaviour, physiological function and the influence of these factors on individual survival remain poorly understood. In particular, it is unclear as to how trade‐offs between these factors are optimised in wild, free‐ranging species.
2. We combined temperature‐sensitive radio transmitters and accelerometers to measure in situ body temperatures and field‐based thermal locomotor performance, estimating thermal optimum and maximum performance. This allowed us to quantify the effectiveness of thermoregulation in the wild and determine whether seasonal trade‐offs in thermoregulatory behaviour shape thermal performance and influence survival in the Australian central bearded dragon (*Pogona vitticeps*).
3. Lizards adjusted their behaviour to maintain optimal body temperatures, achieving greater thermoregulatory precision in spring and summer when environmental costs of thermoregulation were low, but reducing that precision in winter when costs were higher. Activity time and maximum locomotor performance were higher during seasons when thermoregulatory precision was high.
4. Maximum locomotor performance in the field was a strong predictor of survival, regardless of sex, even though survival probabilities were higher in males than females. Higher locomotor performance was associated with increased mortality risk, but survival was not influenced by activity levels or thermoregulatory indices.
5. These findings highlight the complex trade‐offs that ectotherms must navigate to balance behavioural thermoregulation and survival. Our data demonstrate the important influence of seasonal and sex‐specific variation on behaviour and fitness‐related outcomes. Interpreting field‐derived thermal performance curves alongside laboratory measures is crucial for distinguishing ‘true’ physiological capacity from the integrated ecological contexts that shape performance and fitness in nature. Such insights are vital for predicting how ectotherms may respond to future climate warming.

## Graphical abstract

We show higher field‐measured locomotor performance increases mortality risk in free‐ranging lizards, challenging assumptions from laboratory‐based thermal performance studies. Our approach combines in situ physiology with survival data, offering new insights into behavioural trade‐offs and fitness under natural conditions.

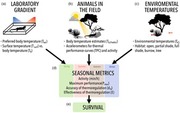

## 1 INTRODUCTION

In meeting competing demands on their time, animals must balance the costs and benefits of various behaviours to maximise their fitness (Huey & Slatkin, 1976). Energy expended in undertaking those behaviours needs to be weighed against fitness gains, where trade‐offs are inevitable and manifest in such contexts as optimal foraging behaviour, investment in mating displays, territorial defence, migration and other allocations of time and energy (Boyd & Hoelzel, 2002; Brown et al., 2018; Campos‐Candela et al., 2019; Huey & Slatkin, 1976). A clear understanding of these trade‐offs may reveal the evolutionary forces that shape various ecological strategies.

Ectotherms rely on external thermal sources to regulate their body temperature (Huey, 1982), a strategy that carries both benefits and constraints (Huey & Slatkin, 1976). Habitat variability often restricts many ectothermic species to narrow thermal margins, requiring behaviours such as shuttling between sun and shade (Huey, 1991). However, some ectotherms inhabit highly variable environments and maintain broader thermal ranges (Huey & Slatkin, 1976; Woods et al., 2015). Although such behaviours help maintain optimal temperatures, they may divert time from mating, foraging and other important activities (Angilletta et al., 2002; Porter et al., 1973; Van Damme et al., 1991). Further, the energy costs of active thermoregulation vary depending on environmental conditions, and these costs can compromise fitness‐related traits (Herczeg et al., 2008; Kearney et al., 2009; Sears & Angilletta Jr., 2015). The presumed links between body temperature and fitness underpin much of thermal ecology, as accurate thermoregulation can confer performance advantages, including enhanced digestion for growth or sprint speed to evade predators (Angilletta, 2009; Angilletta et al., 2002; Pearson & Warner, 2018). However, when access to resources (e.g. food, water, mates) is time‐limited, the benefits of maintaining optimal temperatures must be balanced against trade‐offs such as increased predation risk and additional energy expenditure (Orrell et al., 2004; Skelly, 1994). According to the cost–benefit model of thermoregulation (Huey & Slatkin, 1976), thermoregulation should be more precise when benefits are high and costs are low. Understanding how ectotherms navigate these trade‐offs is crucial for predicting how individuals balance predation risk, energy demands and other constraints. Quantifying these trade‐offs can provide valuable insights into the mechanisms that influence individual growth, reproduction and survival (Chan et al., 2024; Sears et al., 2016).

Variation in environmental conditions, particularly seasonal fluctuations, drives the thermoregulatory decisions that ectotherms must navigate to obtain and maintain optimal body temperatures in the wild (Giacometti et al., 2024). These fluctuations include not only temperature changes but also shifts in water balance, food availability, predation pressures and interactions with conspecifics (Huey & Pianka, 1977; Leith et al., 2024). Seasonal shifts alter the physical and thermal landscape, affecting the availability of suitable microhabitats and thermal refuges in either positive or negative ways (Sears & Angilletta Jr., 2015). For example, in high‐cost environments where ectotherms must expend more time and energy moving between microhabitats to optimise body temperature, individuals may grow more slowly due to energy diverted to thermoregulation (Brewster et al., 2013) or experience increased predation risk due to conspicuous behaviours to regulate body temperature (Basson et al., 2017). Quantifying the behavioural responses to environmental fluctuations can help determine the physiological trade‐offs that may influence survival (Chan et al., 2024).

Life‐history theory for ectotherms explicitly predicts trade‐offs between survival, growth and reproduction, such that investment in one trait reduces the resources available for others, ultimately influencing fitness (Brown et al., 2018; Roff & Fairbairn, 2007; Stearns, 1989). Specifically, increased reproductive effort often incurs direct costs to individual survival due to either heightened energy demands or increased predation risk (Roff et al., 2006; Stearns & Koella, 1986). Thermoregulatory behaviours can mediate these trade‐offs by altering energy allocation strategies, as ectotherms facing seasonal environmental changes must carefully balance the energy costs of active thermoregulation against reproductive investment (Alujević et al., 2023; Calsbeek & Sinervo, 2007; Huey & Slatkin, 1976). Consequently, behavioural decisions around thermoregulation can directly influence the survival–reproduction dynamic and have implications for lifetime fitness (Roff et al., 2002). These life‐history trade‐offs are central to understanding how ectotherms optimise their physiological performance through thermoregulatory strategies.

Heliothermic lizards primarily use behavioural strategies, such as seeking heat and adjusting posture, but can also employ physiological mechanisms like vasoconstriction, panting or colour change to thermoregulate (Huey, 1982; Porter et al., 1973; Smith et al., 2016). The physiological outcomes of these behaviours can be measured using thermal performance curves (TPCs) that assess how ectotherms perform across a range of environmental temperatures. Interpreting their parameters (e.g. critical limits, thermal optimum and maximum performance) in terms of fitness involves linking key curve parameters to survival, growth and reproduction (Huey & Stevenson, 1979). The parameters commonly derived from thermal performance curves, such as thermal optimum and performance capacity at specific temperatures, are correlated to individual survival or other fitness proxies (Angilletta, 2009; Christian & Tracy, 1981; Gilbert & Miles, 2017; Pearson & Warner, 2018). However, TPCs are typically measured under controlled laboratory conditions where variability in temperature, predation and food availability are minimised or eliminated (Albuquerque et al., 2023; Angilletta et al., 2002; Wild & Gienger, 2018). This disconnect contributes to a broader knowledge gap regarding how laboratory‐derived metrics translate into meaningful ecological outcomes for individuals in natural environments (Irschick & Losos, 1998; Husak & Fox, 2006; Warner & Andrews, 2002). Often, it is challenging to accurately measure individual survival in field settings owing to the small size of heliothermic lizards or the rarity of capturing predation events in situ. As a result, survival in lizards is typically inferred from coarse recapture intervals (Gilbert & Miles, 2017; Husak, 2006), which may miss fine‐scale, seasonal mortality patterns. Field‐based studies that continuously track individuals and directly link thermoregulatory behaviour or thermal performance to survival are essential for understanding whether and how laboratory‐based metrics translate to fitness in natural environments.

**FIGURE 1:**
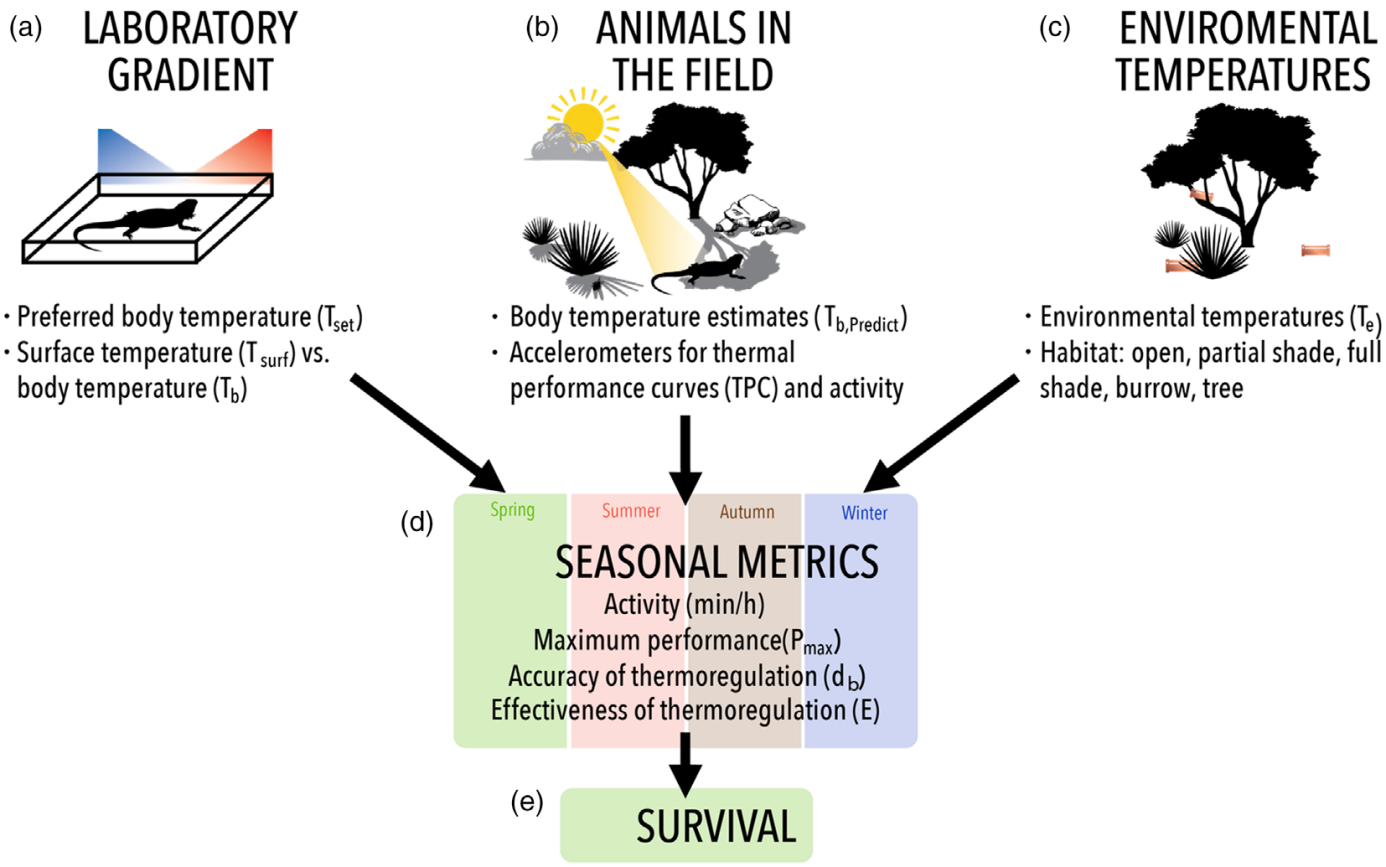
The comprehensive workflow of the experimental design aimed at identifying trade‐offs in thermoregulation and their implications for survival. Laboratory thermal gradient experiments (a) were used to measure the preferred body temperature (*T*_set_) and assess the relationship between surface temperatures (*T*_surf_) recorded with accelerometers and internal body temperatures, enabling the prediction of body temperatures in the field (*T*_b,Predict_). Seasonal thermoregulation and field performance metrics were evaluated using accelerometers (b). Copper pipes were placed in various microhabitats to characterise the thermal environment (*T*_e_) available to lizards in the field (c). Metrics derived from experiments were then compared across seasons (d) and then used as covariates to understand their impact on survival (estimated with known‐fate models) during the spring season (e) when predation pressures are highest for this species.

Using field‐based measurements, we examined how survival relates to common thermal biology metrics (thermoregulatory behaviour and thermal performance curves) in the Australian central bearded dragon (*Pogona vitticeps*). Previous laboratory work with *P. vitticeps* has shown their thermoregulatory behaviours align with the cost–benefit model of thermoregulation (Cadena & Tattersall, 2009). Yet, it is unknown how these behaviours manifest in nature, nor do we understand their fitness outcomes in the wild, free‐ranging individuals. Here, we used temperature‐sensitive radio transmitters equipped with accelerometers to quantify activity and body temperature in the wild (Figure 1), allowing us to generate field‐based thermal performance curves. Unlike traditional laboratory thermal performance curves, which estimate the direct effects of body temperature on performance under controlled conditions, our field‐based approach captures performance variability under realistic ecological conditions, accounting for additional factors that influence performance. We integrated body and environmental temperature measurements to estimate if changes in seasonal thermoregulatory behaviours aligned with predictions of the cost–benefit model of thermoregulation benefit (Figure 1d). Together, these approaches enabled us to examine how aspects of thermal performance curves and thermoregulatory behaviours influence survival (Figure 1e) during the reproductive season (spring) when predation pressures are highest (Wild et al., 2022). Our goal is to understand how thermoregulatory behaviours and thermal performance curves influence survival in heliothermic lizards in situ, providing insight into the cost–benefit model of thermoregulation.

## 2 MATERIALS AND METHODS

### 2.1 Preferred body temperature estimation (*T*_set_) and body temperature calibration

Preferred body temperature (*T*_set_) trials were conducted on adult *P. vitticeps* (*n* = 20; 10 male and 10 female; mean mass = 378.57 g) that were either captured from the study site or captive‐bred descendants of wild‐caught lizards from the study region (see Section 2.2 for region description). Trials were conducted in a temperature‐controlled (20°C) room where internal body temperatures were measured using surgically implanted temperature loggers (iButton® model DS1921G; accuracy ±1°C) recording every 2 min while lizards moved along a laboratory thermal gradient (Figure 1a). The thermal gradient (5.0 m L × 1.0 m H × 2.0 m W) was heated with a series of ceramic heat lamps placed above the gradient and achieved continuous temperatures that ranged from 20°C to 40°C. The thermal gradient contained sand (15 cm depth) and fluorescent lighting that was on a 12 h on/off cycle. Implanted iButtons are a commonly used technique for larger‐bodied reptiles and are considered a best practice for the continuous study of thermal biology of reptiles (Taylor et al., 2021). Postabsorptive lizards were then allowed to recover for a minimum of 48 h before being placed in the thermal gradient and given 12 h to acclimate before initiating measurements. Body temperature recordings used for analysis included only those after the acclimation period. The preferred body temperature was defined as the bounds of the interquartile range of body temperature in the thermal gradient (Hertz et al., 1993). Linear models were used to determine differences in *T*_set_ bounds between sexes.

To predict internal body temperature using external body temperatures (‘surface temperatures’) in field settings, we examined the relationship between body temperature and surface temperature in a subset of captive animals measured in the indoor thermal gradient (*T*_b,Predict_; Figure 1a,b). This subset was equipped with a Pinpoint Beacon 250 transmitter (Lotek Ltd., Havelock North, NZ) that was placed in a custom‐fit backpack harness (Wild et al., 2022). Each transmitter (Pinpoint Beacon 250) and ibutton package for this laboratory experiment weighed (11 g total) less than 5% of the mass of the lizard. Each Pinpoint Beacon 250 housed a temperature data logger that recorded surface temperature every 2 s, which was averaged every 2 min to pair with body temperature with iButton. Gradient methods followed the same protocol described above. The relationship between body and surface temperature was estimated using linear regression and paired t‐test (surface vs. internal temperature at each time point) to examine the degree to which surface temperature underestimated or overestimated body temperature. The equation from the linear regression between body and surface temperature was used for *T*_b,Predict_ correction.

### 2.2 Field study area and radiotelemetry

Field work for this study was conducted in a 140 km^2^ nature reserve (Bowra Wildlife Sanctuary) near Cunnamulla Queensland, Australia. Adult *P. vitticeps* were captured opportunistically and tracked continuously between October 2018 to September 2019. Each lizard was fitted with a Pinpoint Beacon 250 using the same custom‐fit backpack harness used in the *T*_b,Predict_ experiment. Each unit housed a GPS logger, a single‐stage VHF transmitter (150–151 Hz), a temperature data logger and a 2‐axis accelerometer. Phenotypic sex was determined using hemipenile eversion. During the reproductive season (spring), females were palpated biweekly when transmitters were replaced, and gravid females were excluded from all analyses. For further information on lizard collection, site description or radio telemetry, see Wild et al. (2022).

### 2.3 Field predicted body temperature, environmental temperature and thermoregulatory strategy

Temperature dataloggers in the Pinpoint Beacon 250 measured the range of temperatures that lizards experience in the wild. Loggers recorded a surface temperature (°C) every 2 s, and this was averaged over 1 min. The surface and body temperature correction was applied (Figure 1a) to estimate field body temperature (*T*_b,Predict_).

Environmental temperatures available to animals within the landscape (*T*_e_) were estimated using physical models (Bakken & Gates, 1975) that were the same length and width as an average lizard. Models were constructed of hollow copper pipes (40.0 mm outside diameter, 1.22 mm wall thickness, 250 mm length) with an iButton suspended in the centre (Figure 1c). These models were validated by comparison with fresh lizard carcasses that had implanted iButton dataloggers recording internal body temperature (*see* SI for calibration methods), but were not designed to estimate true operative temperatures based on instantaneous heat flux equilibrium (i.e. operative temperature). Copper models were deployed from October 2018 to September 2019 and recorded environmental temperature (*T*_e_) every 1 h. Copper models were placed in five microhabitat categories: full shade (*n* = 10), partial shade (*n* = 10), open (*n* = 10), tree (*n* = 12) and burrow (*n* = 8; see Table S1 for definitions of microhabitat categories). Microhabitats accessible to *P. vitticeps* were considered when positioning each model (see Supporting Information). Mean *T*_e_ measurements were calculated for each hour between 05:00–21:00 to obtain a measure of the environmental temperature of the habitat available to *P. vitticeps* for any given hour during the study. We assumed males and females experienced the same distribution of thermal microhabitats.

Metrics of thermoregulation were quantified using laboratory preference range (*T*_set_) and hourly measurements of environmental (*T*_e_) and body temperature (*T*_b,Predict_) in the field. The accuracy of thermoregulation (*d*_b_) was defined as the overall mean deviations of body temperatures from the thermal preference range (calculated using sex‐specific *T*_set_ values). Similarly, the average thermal quality of the habitat (*d*_e_) was assessed by estimating the overall mean deviations of environmental temperatures from the thermal preference range for each individual copper model in each habitat (Hertz et al., 1993). These metrics were calculated hourly between 05:00–21:00 h across the year. The hourly effectiveness of thermoregulation (*E*) for each individual lizard was then calculated using *d*_b_ and *d*_e_ with the following equation:

$$E=1-(\bar{d_{b}}/\bar{d_{e}})$$

where *E* is expressed as a ratio generally ranging from 0 to 1, and over bars indicate mean deviations of body and environmental temperature. An *E* of 1 reflects highly effective thermoregulation, meaning that an animal maintains body temperatures close to its preferred range despite thermal conditions. In contrast, an *E* of 0 indicates that an animal's body temperatures are no better than the surrounding environmental temperatures, consistent with thermoconformity (Hertz et al., 1993). It is possible for *E* to be negative in situations where an individual actively avoids the thermal preference range even though *T*_e_ allows the opportunity for thermoregulation within the thermal preference range. Low *E* values can occur when predators are abundant, food availability is scarce, or during interaction with conspecifics (Christian & Weavers, 1996). All metrics of thermoregulation (*T*_b,Predict_, *d*_b_, *d*_e_ and *E*) were averaged for each individual over the course of each season prior to analysis. For each metric, a linear mixed‐effects model was used to test the effect of season, sex and their interactions, with season and sex as fixed effects and either lizard ID (or model ID) as a random effect.

### 2.4 Activity and thermal performance curves

Activity (min/h) and field thermal performance curves (TPC) were estimated using accelerometry and temperature data provided by the Pinpoint Beacon 250. Accelerometers recorded acceleration on two axes corresponding to X‐heave and Y‐surge at a rate of 6 Hz. Acceleration values were averaged for each axis (1 min) between 05:00 and 21:00 h for each season. Each axis of acceleration was transformed to resultant acceleration (hereafter acceleration, ms^−2^) following manufacturer protocols (see Supporting Information for transformation details). Activity was defined as any change in acceleration from the previous value between samples taken with the accelerometer and calculated as the minutes moved for each hour (min/h). For analysis purposes, activity was log‐transformed (log(*x* + 1)) to deal with the abundant sedentary periods in which individuals did not move (i.e. no changes in acceleration).

Thermal performance curves were constructed using *T*_b,Predict_ and acceleration (ms^−2^) values from accelerometers. Body temperatures (*T*_b,Predict_) were averaged for each 1 min to match the averaged timescale of acceleration data. General additive mixed models (GAMM) were used with *T*_b,Predict_ as the predictor and acceleration (i.e. performance) as the response variable. Performance for TPC was defined as the 95th percentile of acceleration at each 1°C. This allowed for the characterisation of the upper capacity for movement while avoiding the influence of outliers resulting from the many sedentary periods. This also ensured that we captured the highest possible value, allowing for the closest comparison to laboratory TPCs. The package mgcv was used for cubic spline rolling average regression for all GAMM (Wood, 2017). Model selection, fitting and validation followed Zuur et al. (2009). The most inclusive GAMM included (in addition to temperature) season, sex and their interaction as fixed effects, and individual as a random effect (modelled as a smoothed cubic spline). The maximum predicted acceleration (ms^−2^) from GAMM fit was defined as *P*_max_ and the temperature associated with *P*_max_ was defined as *T*_opt_ (Angilletta, 2009). For each TPC metric (*P*_max_ and *T*_opt_), a linear mixed‐effects model was used to test the effect of season, sex and their interactions, with season and sex as fixed effects and lizard id random (repeated) effect. The *gam.check()* function from the package mgcv was used to examine model convergence, gradient range, Hessian matrix characteristics and basis dimension checking results.

### 2.5 Estimating survival

Maximum likelihood survival probabilities were estimated using known‐fate models (White & Burnham, 1999). Known‐fate models assume perfect detection (sampling probability = 1), meaning that the fate (alive or dead) of each radio‐tagged animal is known with certainty at each sampling occasion. Thus, survival is modelled using a product of binomial likelihoods, where animals not confirmed dead (i.e. carcasses not recovered) are treated as alive or censored (due to loss of telemetry gear or transmitter failure) but never assumed dead. Parameter estimates derived from known‐fate models were then used to determine the extent to which thermal or performance estimates could predict an individual's survivorship in the field (Figure 1e). Survival was determined from daily telemetry surveys from Spring 2018, during which deaths were recorded based on recovered carcasses. Animals were only classified as dead if carcasses were physically recovered. In cases where the cause of mortality could be inferred, depredated individuals exhibited extensive fresh injuries to the body and transmitter, likely from a raptor or mammalian predator; individuals without clear signs of predation were noted separately. Spring was used for this analysis because movement rates were elevated and most variable among individuals, and mortality rates were highest during this period (Wild et al., 2022), providing the best opportunity to link variation in thermal and performance estimates with survival outcomes. AICc was used to correct for small sample sizes when estimating survivorship using known‐fate models during the spring season, and models with ΔAICc of <2.0 were considered to have support. The analysis started with a fully saturated model in which survival probability during the spring was dependent on movement (min/h), accuracy of thermoregulation (*d*_b_), effectiveness of thermoregulation (*E*) and maximum performance (*P*_max_) as covariates, then a series of reduced‐parameter models were fitted where sex was included (or removed) as an interaction.

### 2.6 Statistical analysis

Statistical analyses were performed using the R environment ver. 4.1.0 and survivorship estimates using the program MARK (White & Burnham, 1999). All analyses were tested for normality. If data did not fit normality assumptions, the appropriate transformation was applied to achieve normality. Seasonal periods were spring, summer, autumn and winter for all analyses. Statistical significance was accepted at the *p* < 0.05, and if results were significant, they were followed with the appropriate post hoc test. Data collection for this project was performed under UC Animal Ethics approval AEC 17‐13.

## 3 RESULTS

### 3.1 Preferred body temperature estimation (*T*_set_) and body temperature calibration

Females consistently had higher preferred body temperatures than males. This was observed in the 75% quantile measurements, with females at 33.8 ± 0.92°C and males at 29.0 ± 0.92°C (*F*_1,18_ = 4.78; *p* < 0.05). Similarly, in the 25% quantile measurements, females had estimates of 27.0 ± 0.46°C, while males had 25.5 ± 0.46°C (*F*_1,18_ = 4.77; *p* < 0.05).

There was a strong relationship between laboratory body temperature and surface temperature (16,938 paired measurements were recorded for 10 individuals; *R* ^2^ = 0.94; *F*_1,16,937_ = 2,469,723; *p* < 0.01). Surface temperature slightly overestimated body temperature by 0.12 ± 0.01°C (paired *t* = 12.21; df = 16,938; *p* < 0.01), so body temperature estimates (*T*_b,predict_)were corrected from surface temperatures using the linear regression results:

$$T_{b,\text{Predict}}=1.770+\left(T_{\text{surf}}\times 1.058\right).$$

### 3.2 Thermoregulation in the field

**FIGURE 2:**
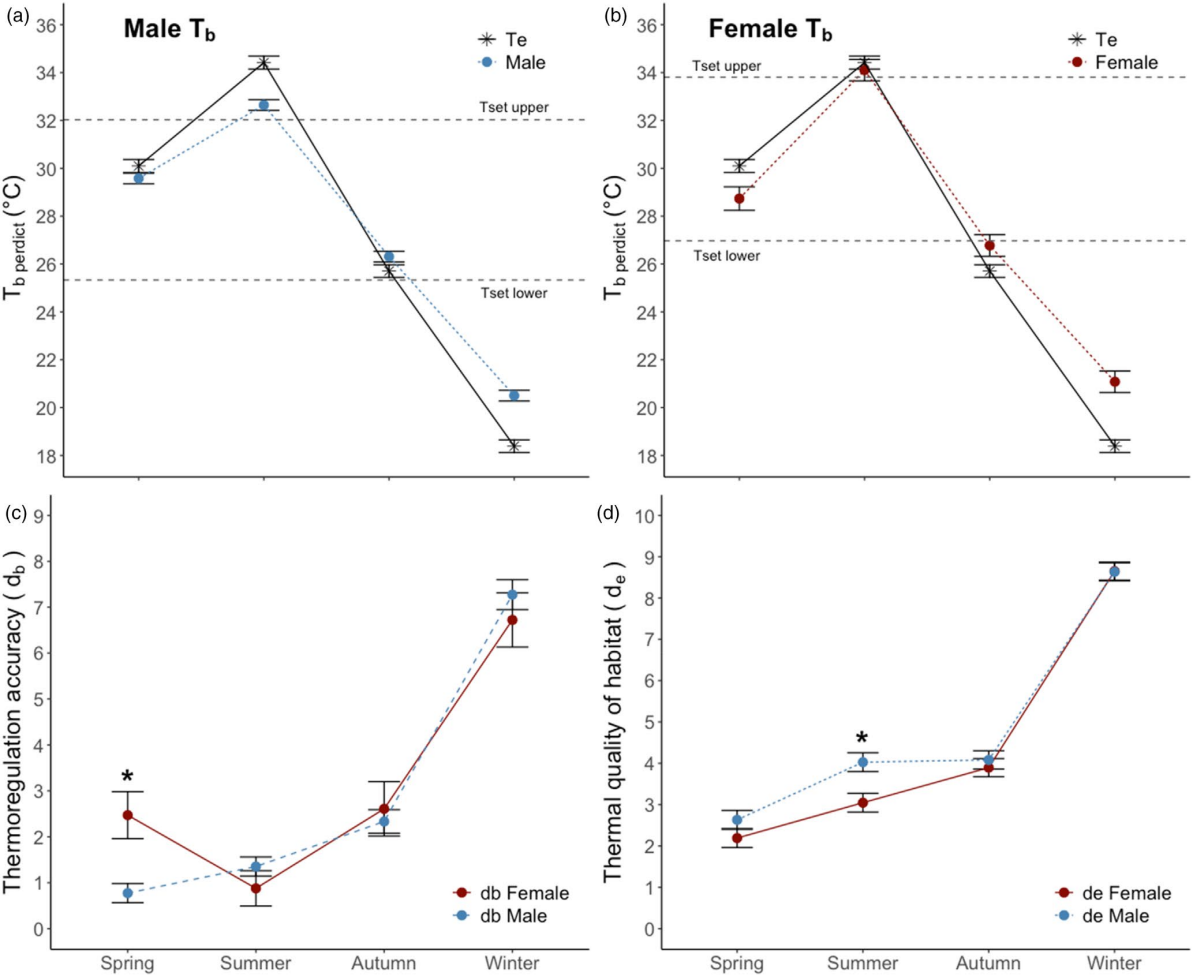
Mean seasonal environmental temperature (*T*_e_), thermal preference (*T*_set_) and predicted body temperature (*T*_b,Predict_) for male (a) and female (b) *Pogona vitticeps*. Accuracy of thermoregulation (*d*_b_) between sex (c), where low *d*_b_ denotes body temperature closer to thermal preference. The thermal quality of habitat (*d*_e_), measured using copper models that account for sex differences in thermal preference (d). Low *d*_e_ values indicate more environmental temperatures fell within *T*_set_ (i.e. favourable thermal environment). Error bars for all panels are ±1 standard error of the mean. The asterisk symbol indicates a significant difference (*p* < 0.01) when comparing mean differences between sexes for that season.

Thermal‐sensitive accelerometers were placed on 40 individual *P. vitticeps* (male: *n* = 32; female: *n* = 8) that were tracked between Spring 2018 and Winter 2019. For a subset of these individuals (*n* = 8), we validated our *T*_b,predict_ estimates by concurrently recording core body temperature with implanted iButtons and found they closely approximated actual core temperature (*r* ^2^ = 0.86, Figure S1). There were differences in seasonal body temperatures (*T*_b,Predict_) (*p* < 0.01) and a season × sex interaction (*p* < 0.01; Table S2), but for sex alone, there were no differences (*p* = 0.40). Least squares estimates indicated significant seasonal differences in *T*_b,Predict_ (Table S3), with the highest values in summer (33.4 ± 0.25°C), followed by spring (29.2 ± 0.27°C), autumn (26.5 ± 0.25°C) and winter (20.8 ± 0.25°C). Least squares estimates for the interaction suggested that differences in *T*_b,Predict_ between the sexes were only observable during the summer (Figure 2a,b), where females selected higher body temperatures than males. There were no detectable differences in *T*_b,Predict_ during other seasons (Table S4).

Mean *T*_e_ was different across all seasons (*F*_3,329,321_ = 371.03; *p* < 0.01), with higher temperatures observed in spring and summer, and lower temperatures in autumn and winter (Figure 2a,b). Season and the interaction between sex and season had an effect on the accuracy of thermoregulation (*d*_b_), but there was no overall effect of sex on *d*_b_ estimates (Table S2). Males thermoregulated more accurately (i.e. low *d*_b_) than females during spring, and there were no differences during the other seasons (Figure 2c; *p* < 0.05). Season, sex and the interaction had an overall effect on the thermal quality of the habitat (*d*_e_, Table S2). The thermal environment was more favourable (i.e. lower *d*_e_) for females than males during the summer (Figure 2d; *p* < 0.05) because females had a higher T_pref_ range than males.

**FIGURE 3:**
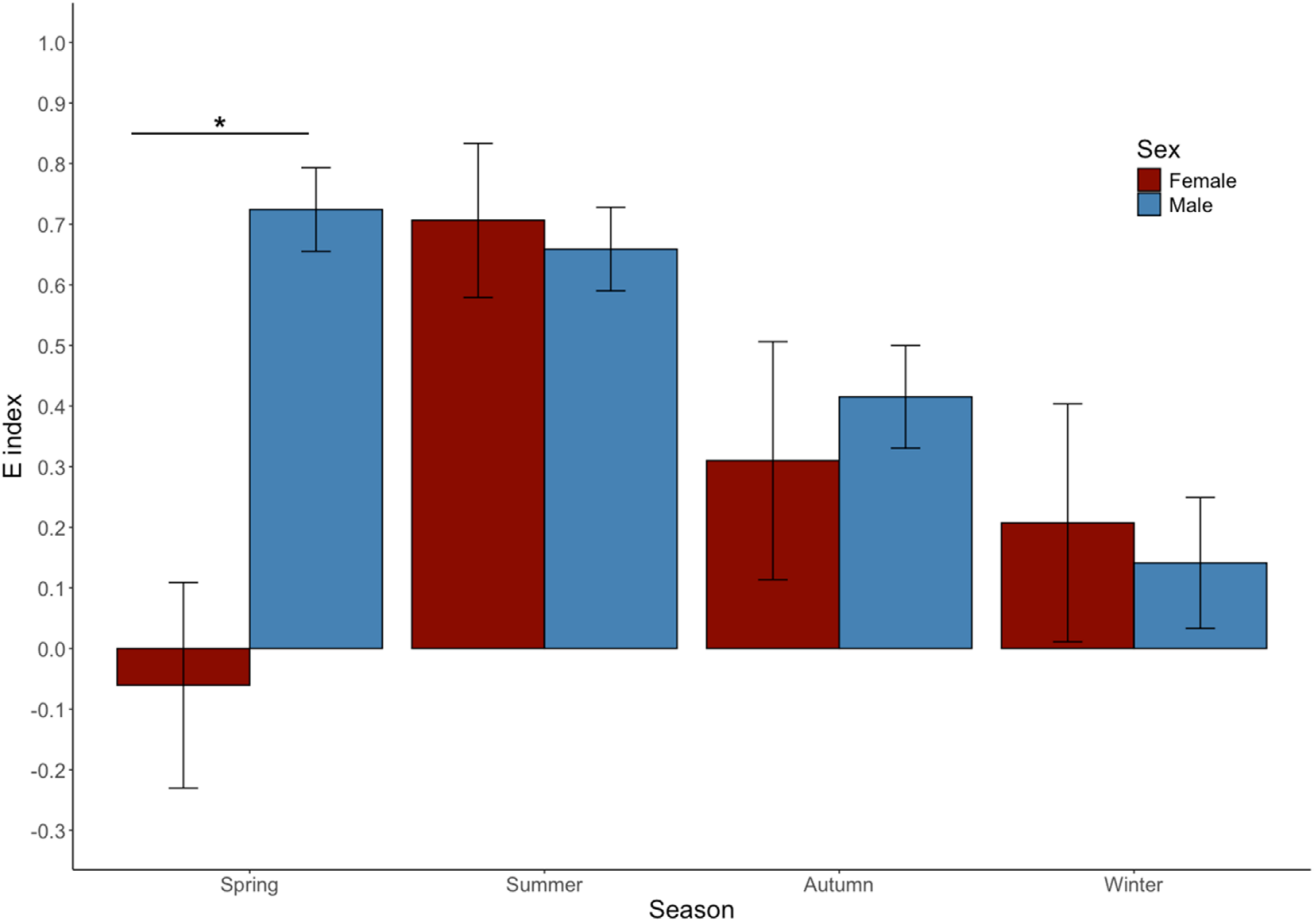
Effectiveness of thermoregulation (*E* index) by sex and season in *Pogona vitticeps*. *E* values approaching 0 indicate thermoconformity (body temperatures closely track environmental temperatures), while values approaching 1 indicate highly effective thermoregulation (body temperatures maintained near preferred values despite environmental variation). Data are means accounting for all individuals for each season. Error bars indicate ±1 standard error of the mean. The asterisk symbol denotes a significant difference (*p* < 0.01) between sex when comparing mean differences for that season.

The effectiveness of thermoregulation (*E*) was influenced by season, but the effect of season was different between sexes (Table S2; Figure 3). In the spring season, females were not effective thermoregulators (i.e. low *E*), whereas males were effective thermoregulators (i.e. high *E*). Both male and female lizards were effective thermoregulators during summer (Figure 3). However, in the autumn and winter, males and females were less effective at thermoregulating (Figure 3). Overall, males were more effective thermoregulators (0.48) than females (0.29; Table S2; *p* = 0.05).

### 3.3 Seasonal activity and thermal performance curves

**FIGURE 4:**
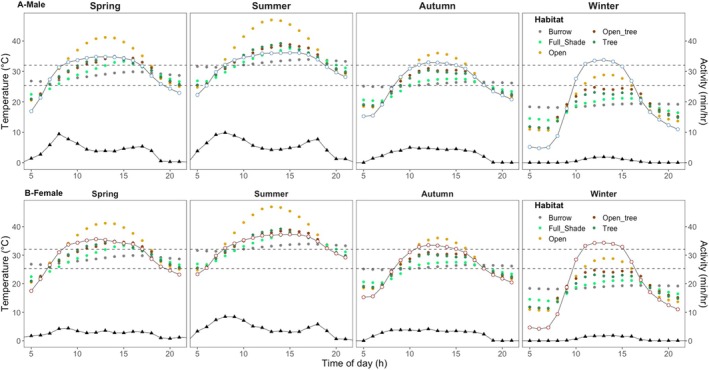
Mean predicted body temperatures (lines with circles) and activity levels (lines with triangles) for male (a) and female (b) *Pogona vitticeps* by season and time of day. The dashed line represents their preferred body temperature range for each sex. Coloured circles indicate mean environmental temperatures for different habitat types, measured using copper models.

A total of 6,858,857 raw acceleration data points were collected on male (*n* = 32) and female (*n* = 8) *P. vitticeps*. Average movement varied across the season (*F*_3,81_ = 9.25; *p* < 0.01), but there were no differences between sexes (*F*_1,68_ = 0.23; *p* = 0.63) or the interaction (*F*_3,81_ = 0.29; *p* = 0.83). Overall activity was highest in the summer and lowest in the winter (Figure 4; Table S4).

**FIGURE 5:**
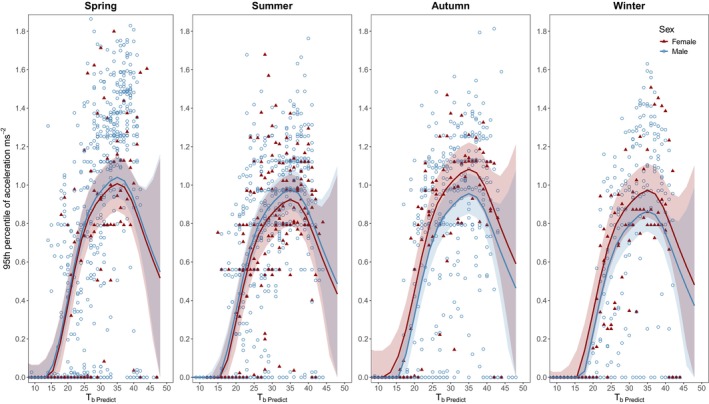
Thermal performance curves of free‐ranging *Pogona vitticeps* across season and sex. The data were obtained from the top‐performing generalised additive mixed models (GAMM) presented in Table S5. Each data point represents the average performance (95th percentile of acceleration) at a given temperature for all individuals in each season and sex. Bands around lines are 95% CI of model fit.

The top candidate GAMM model for field thermal performance curves (ΔAIC score = 0.00) accounted for season, sex and their interaction allowing for random intercept and smoothed spline per individual and explained 71% of the total deviance (Figure 5; see Section S5 for other model comparisons). Season (*F*_3,88_ = 190.62; *p* < 0.01) and the interaction between sex and season (*F*_3,88_ = 143.08; *p* < 0.01) had an overall effect on the maximum performance, but there was no effect on sex alone (*F*_1,90_ = 0.34; *p* = 0.56). Maximum locomotor performance (*P*_max_) was highest in spring, whereas winter had the lowest values of other seasons (*p* < 0.05; Table S6). Females exhibited higher *P*_max_ values in autumn and winter than in other seasons, and males demonstrated higher values in spring and summer than in other seasons (Table S7). The average thermal optimum (*T*_opt_) temperature (mean ± SE) was 36.6 ± 0.24°C. There were no differences in *T*_opt_ across seasons (*F*_3,88_ = 0.24; *p* = 0.63), between sex (*F*_1,90_ = 0.57; *p* = 0.64) or their interaction (*F*_3,88_ = 1.79; *p* = 0.64).

### 3.4 Applying metrics of thermoregulation, activity and performance to survival

**FIGURE 6:**
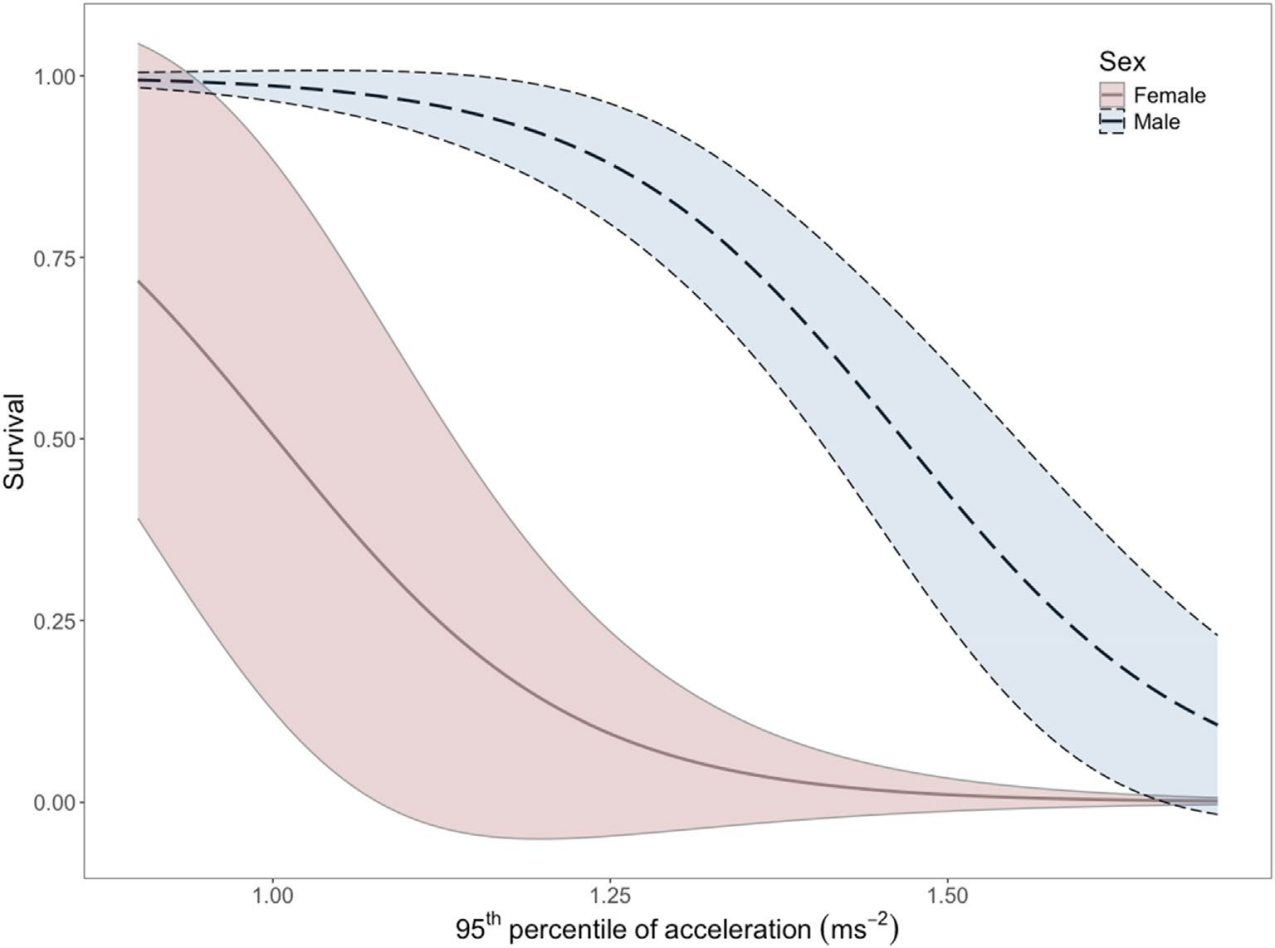
Survivorship as a function of the maximum performance (*P*_max_) for free‐ranging male and female *Pogona vitticeps* in spring (September–November). Data are extracted from the top‐performing known‐fates survival model in Program MARK that accounted for sex (Table S8). Lines represent the predicted mean survival for each sex, and bands indicate 95% CI.

Twenty‐seven lizards were tracked during the spring, eight of which died during this period. Seven mortalities showed signs of predation, with extensive injuries consistent with raptor or mammalian predation. One individual showed no evidence of predation, as indicated by the absence of injuries or disturbance to the body. Survival probabilities (mean ± SE) were higher for males (0.75 ± 0.08) than females (0.33 ± 0.20). The top competing model accounted for sex and maximum performance (Figure 6; Table S8). There was a distinct pattern between performance and survival for both sexes, where individuals with lower maximum performance had higher survival rates compared to those with higher performance. This decline happened at lower levels of *P*_max_ in females than in males, such that a given *P*_max_ was associated with lower survival in females than males (Figure 6).

## 4 DISCUSSION

In the context of the cost–benefit model of thermoregulation (Huey & Slatkin, 1976), our study provides important insights into the trade‐offs between thermoregulation, locomotor performance and survival in ectotherms. Previous studies have suggested that increased locomotor activity can elevate predation risk (Vitt & Congdon, 1978) and that individuals with higher locomotor performance may incur greater costs associated with reproduction or survival (Cooper et al., 1990; Padilla Perez & Angilletta Jr., 2022; Vitt & Price, 1982). Using telemetry and temperature‐sensitive accelerometry, we generated the first in situ thermal performance curves derived from accelerometers for an ectotherm, providing a rare examination of thermoregulatory strategies and their associated seasonal trade‐offs in the field. Notably, our findings reveal that maximum performance correlates positively with mortality risk for both males and females, with this effect being more pronounced in females during the reproductive season (spring). While survival during spring does not fully capture lifetime reproductive success, it remains a critical fitness‐related trait, as individuals who die would have no further reproductive opportunities. Our field observations indicate that predation was likely the primary cause of death for lizards, consistent with predation observations documented in this same population (Wild et al., 2022). Regardless of the exact cause of death, maximum locomotor performance was strongly linked to mortality risk. These results challenge the traditional view that higher locomotor performance within the thermal optimum will enhance fitness outcomes in the field (Calsbeek & Sinervo, 2007; Christian & Tracy, 1981; Gilbert & Miles, 2017).

Interpreting the parameters of thermal performance curves (TPCs) derived from field data requires careful consideration of their conceptual differences from laboratory‐based TPCs. Laboratory‐based TPCs often isolate ‘true’ physiological performance metrics by directly stimulating animals to perform (e.g. forced running, biting) while controlling tightly for extrinsic environmental variables (Angilletta, 2009; Taylor et al., 2021). In contrast, field‐based TPCs inherently capture the integrated ecological contexts—including predation risk, resource availability and environmental variability—which shapes fitness‐relevant behaviours and traits (Childress & Letcher, 2017; Nowakowski et al., 2020). For instance, optimal temperatures (*T*_opt_) in field settings do not merely represent physiological peaks but correspond to conditions where animals maximise fitness components such as survival, growth and reproduction (Clusella‐Trullas et al., 2011; Kingsolver & Gomulkiewicz, 2003). Performance measured as maximum movement capacity in the field might reflect behavioural choices influenced by multiple ecological factors beyond temperature alone (Alujević et al., 2023; Childress & Letcher, 2017). Future studies could benefit from comparing field‐derived thermal performance curves with laboratory‐based estimates.

Contrary to previous studies that have linked maximum locomotor performance (e.g. sprint speed measured in controlled laboratory conditions) to increased survival in the wild (Christian & Tracy, 1981; Gilbert & Miles, 2017; Pearson & Warner, 2018), we found that higher maximum performance was associated with decreased survival in the wild. Our findings contrast with previous work, which demonstrates positive associations between thermoregulatory accuracy, the thermal quality of the environment and fitness‐related traits such as survival and reproductive success in the field (Alujević et al., 2023; Calsbeek & Sinervo, 2007). For instance, Calsbeek and Sinervo (2007) experimentally improved the thermal environment of territories and observed increased juvenile survival due to more efficient thermoregulation. Alujević et al. (2023) demonstrated that higher thermal quality of territories is associated with enhanced reproductive behaviours and greater reproductive success. Our field‐based observations, in contrast, suggest that high‐performing (*P*_max_) individuals may engage in conspicuous or risky behaviours (Horváth et al., 2024), thereby increasing predation risk due to heightened visibility or expanded home ranges in predator‐rich areas (Skelly, 1994; Ward‐Fear et al., 2018). Our findings show that in natural settings, high *P*_max_ may not universally confer survival advantages and, under certain ecological contexts, can be associated with elevated mortality risk. We acknowledge the limitations of our modest sample size for survival (*n* = 27), but similar cohorts are not uncommon in field‐based telemetry studies (e.g. McIntyre et al., 2006, Golden Eagle [*n* = 22]; Olson et al., 2013, Hellbenders [*n* = 21]; Goetz et al., 2021, Brown Treesnake [*n* = 30]; Ferronato et al., 2016, Eastern Long‐necked turtle [*n* = 46]). These data provide high‐resolution ecological information despite increased uncertainty in parameter estimates.

Outside of the reproductive season for females, we found that activity patterns, thermoregulation metrics and maximum performance followed general predictions of the cost–benefit model of thermoregulation (Huey & Slatkin, 1976). We observed that during winter, when thermoregulation is more challenging due to lower ambient temperatures and limited time to achieve thermal preference, there was a decline in both the accuracy and effectiveness of thermoregulation. These declines coincided with decreases in other physiological traits that are temperature‐dependent, such as maximum locomotor performance (ms^−2^) and fine‐scale activity (min/h). Conversely, during the summer, the accuracy and effectiveness of thermoregulation were high, which corresponded with increased activity levels and maximum locomotor performance. These seasonal trade‐offs demonstrate the dynamic balance that lizards must maintain while accounting for the energy trade‐offs of thermoregulation (Angilletta & Sears, 2000; Sears & Angilletta Jr., 2015; Vickers et al., 2011). Although the effectiveness of thermoregulation was not associated with survival in our study, this metric has been shown to have direct consequences for growth, reproductive success and even survival in other lizards (Basson et al., 2017; Brewster et al., 2013; Sears et al., 2016).

Sex differences in ectotherm thermal biology, largely documented from laboratory data or short‐term field manipulations, show that males and females can exhibit distinct thermoregulatory behaviours and thermal performance traits associated with different ecologies and reproductive strategies (Beal et al., 2014; Lailvaux et al., 2003; Ortega et al., 2016). However, translating the ecological significance of these results into natural systems remains challenging because continuous field observations are needed to track how seasonality, reproductive demands and species interactions shape thermoregulatory strategies in both sexes (Bodensteiner et al., 2021; Huey & Pianka, 2007; Pottier et al., 2021). There are examples where female lizards exhibit altered thermoregulatory behaviours during reproductive periods, leading to trade‐offs between optimal body temperature maintenance and reproductive or predator‐avoidance strategies (Logan et al., 2021; Ortega et al., 2016). In *P. vitticeps*, females exhibit overall higher energy demands (Wild et al., 2023) and poor body condition during the reproductive season (Wild et al., 2022), which may contribute to the sex‐specific differences in survival. While existing literature emphasises behavioural or physiological distinctions between sexes, few studies have directly linked these thermal strategies to explicit fitness outcomes, such as survival under natural conditions.

Laboratory results in other ectothermic vertebrates suggest limited plasticity in optimal temperatures (MacLean et al., 2019; Pottier et al., 2022; Zhang et al., 2023). Our findings support this pattern, where the field optimal temperature (36.6 ± 0.24°C) remained consistent across sexes and seasons. Constrained thermal optimum suggests that energetically expensive behaviours, like thermoregulation, are necessary to maintain optimal temperatures throughout the year, regardless of environmental changes (Huey et al., 2012; Wild, Huey, et al., 2025; Wild, Roe, et al., 2025). This requirement becomes particularly challenging during energetically demanding periods, such as spring and summer, when heliothermic lizards divert surplus energy reserves towards reproduction (Nagy, 1983). Maintaining a static thermal optimum appears to be crucial for optimal performance, despite the costs associated with thermoregulation (Herczeg et al., 2008; Huey & Slatkin, 1976). These findings demonstrate the trade‐offs involved in maintaining optimal body temperatures, as the energy costs of thermoregulation must be balanced against other physiological needs (Blouin‐Demers & Nadeau, 2005; Vickers et al., 2011).

By using temperature‐sensitive accelerometers in conjunction with surface calibration**s**, we derived predicted body temperature estimates (*T*_b,predict_: 32.7 ± 0.02°C) that closely matched previously published core field body temperatures for this species (34.3 ± 3.75°C: Greer, 1989; 32.9 ± 0.88°C: Melville & Schulte, 2001). However, future studies might benefit from improved operative modelling techniques, such as copper electroforming or 3D printing, which have shown greater accuracy, reproducibility and cost‐effectiveness for quantifying operative temperatures in terrestrial thermal environments (Alujević et al., 2024). Combining basic physiological measurements with thermosensitive accelerometers offers a powerful approach for testing challenging ecological and physiological hypotheses in thermal ecology. New applications of accelerometers, including linking movement data to field energy expenditure (doubly labelled water) and identifying specific behaviours with raw acceleration, provide promising avenues for future research across diverse vertebrate groups (Chakravarty et al., 2019; Garde et al., 2022; Pagano & Williams, 2019). Such approaches will be crucial for understanding how physiological traits vary under field conditions in a warming and increasingly variable climate.

## AUTHOR CONTRIBUTIONS

Kristoffer H. Wild organised the sampling design, collection of materials, laboratory work and figures with the support of John H. Roe, Jonathan Curran, Phillip R. Pearson, Lisa Schwanz, Arthur Georges and Stephen D. Sarre. Kristoffer H. Wild wrote the first draft of the manuscript. Kristoffer H. Wild organised all data curation and final analysis. Comments from John H. Roe, Jonathan Curran, Phillip R. Pearson, Lisa Schwanz, Arthur Georges and Stephen D. Sarre contributed to the final version of the manuscript. All authors agreed on the final approval of the version to be published and agreed that they are accountable for the work they conducted.

## CONFLICT OF INTEREST STATEMENT

The authors declare no conflicts of interest.

## Supporting information


**Table S1:** Microhabitat categories of sun exposure.
**Table S2:** ANOVA table for predicted body temperature (*T*_b,Predict_), accuracy of thermoregulation (*d*_b_), thermal quality of habitat (*d*_e_), and effectiveness of thermoregulation (*E*) for *Pogona vitticeps*.
**Table S3:** Tukey‐Kramer multiple comparisons from *T*_b,predict_ model (Table 2).
**Table S4:** Tukey‐Kramer multiple comparisons of overall seasonal activity rate (min/h).
**Table S5:** General additive mixed‐models for investigating how performance curves varied across season, sex and their interactions for *Pogona vitticeps*.
**Table S6:** Tukey‐Kramer multiple comparisons from the *P*_max_ model that accounted for the season, sex and interaction.
**Table S7:** Tukey‐Kramer multiple comparisons from the *P*_max_ model that accounted for the season, sex and interaction.
**Table S8:** Model comparisons of spring survival probability (*φ*) for *Pogona vitticeps*, depending on sex, movement (min/h), accuracy of thermoregulation (*d*_b_), effectiveness of thermoregulation (*E*), and maximum performance (*P*_max_).
**Figure S1:** Comparison of predicted and core body temperatures of lizards in the field.
**Figure S2:** Environmental temperature range and how *Pogona vitticeps* thermoregulated during the duration of the study.
**Figure S3:** Relationships between maximum performance (*P*_max_), accuracy of thermoregulation (*d*_b_), and efficiency of thermoregulation (*E* index) with minutes active.

## Data Availability

Data, code and additional resources are available on GitHub: https://github.com/kris‐wild/TPC_Survival.git and Zenodo: https://zenodo.org/records/15644678.
