# Supplementary material for: Thermal performance curves, activity and survival in a free‐ranging ectotherm
Source: J Anim Ecol. 2025 Jul 16;94(9):1823–36. doi: 10.1111/1365-2656.70091 (PMC12424282; doi:10.1111/1365-2656.70091)
Supplement: Supplementary file 1 — Table S1: Microhabitat categories of sun exposure. Table S2: ANOVA table for predicted body temperature (T b,Predict), accuracy of thermoregulation (d b), thermal quality of habitat (d e), and effectiveness of thermoregulation (E) for Pogona vitticeps. Table S3: Tukey‐Kramer multiple comparisons from T b,predict model (Table 2). Table S4: Tukey‐Kramer multiple comparisons of overall seasonal activity rate (min/h). Table S5: General additive mixed‐models for investigating how performance curves varied across season, sex and their interactions for Pogona vitticeps. Table S6: Tukey‐Kramer multiple comparisons from the P max model that accounted for the season, sex and interaction. Table S7: Tukey‐Kramer multiple comparisons from the P max model that accounted for the season, sex and interaction. Table S8: Model comparisons of spring survival probability (φ) for Pogona vitticeps, depending on sex, movement (min/h), accuracy of thermoregulation (d b), effectiveness of thermoregulation (E), and maximum performance (P max). Figure S1: Comparison of predicted and core body temperatures of lizards in the field. Figure S2: Environmental temperature range and how Pogona vitticeps thermoregulated during the duration of the study. Figure S3: Relationships between maximum performance (P max), accuracy of thermoregulation (d b), and efficiency of thermoregulation (E index) with minutes active. [file JANE-94-1823-s001.docx]

**Supplement Information**

Surgical protocols:

*Surgical protocols:* Internal body temperature (T_b_) was measured with a surgically implanted Thermochron iButton. To accurately measure internal body temperature (T_b_), a Thermochron iButton was surgically implanted following the surgical techniques outlined by(Koenig et al., 2001). Each lizard (male: n = 10; female: n = 10) was given an inhalant anaesthetic (isoflurane 3 – 5) until the surgical plane of anaesthesia was reached. All iButtons were inserted into the peritoneal cavities by a 2cm incision through the ventral abdominal wall. Following surgery, lizards were allowed 48h to recover from surgery procedures prior to being placed in the gradient and then were placed in the gradient. The first 12h were considered an acclimation period once lizards were placed in the gradients before iButtons began recording T_b_ every 10min.

Body temperature validation in the field

*Field body temperature vs laboratory body temperature adjustment:* A subset of individuals in the field (n = 8) had iButtons that were surgically implanted following the surgical protocols described above. iButtons recorded hourly core body temperatures (T_b,obs_) from January to March 2019. Accelerometers that recorded temperature were placed on these animals so that the laboratory T_b,predict_ adjustment from surface temperatures could be compared to observed field core body temperatures. Comparisons of our body temperature adjustment (T_b,predict_) and body temperature (T_b,obs_) measured with surgically implanted iButtons revealed a close and near one-to-one relationship (regression statistics ± 1 se: slope = 0.82 ± 0.004, intercept = 3.74 ± 0.140, r^2^ = 0.86, N = 6,961, t = 203.87, p < 0.001). It appeared that (T_b,predict_) slightly under predicted core body temperature (Fig S1).

Environmental model calibration:

*Environmental model calibration:* Models were calibrated using a fresh carcasses of *P. vitticeps*, which were placed beside one of the copper models on the ground in partial shade during three sunny days in November 2018. Temperatures were recorded in the carcass and the model every 5min from dawn to dusk. We used a linear regression of the carcass temperature to the model temperature to subsequently correct all records from field-deployed copper models (T_e_).

Accelerometer protocol and TPC analysis:

*Calculation of Resultant Acceleration***:** To calculate the resultant acceleration, we considered only the x and y axes due to the limited acceleration on the z-axis for lizards. Resultant acceleration was computed using the Euclidean norm as follows:

$$Resulant acceleration= \sqrt{a_{x}^{2}+a_{y}^{2}+a_{z}^{2}}$$

where a_x_, a_y_ and a_z_ are the accelerations along the x, y, z axes, respectively. The z-axis was ignored due to limited acceleration on that plane for lizards. This resultant acceleration provides a measure of the overall intensity of movement, integrating the contributions from both axes. This method ensures a comprehensive representation of the lizard's activity based on changes in acceleration.

*Model selection TPC:* Other GAMMs in the series considered all reduced variants of this model. This approach allowed us to compare Akaike Information Criteria (AIC) changes among models and allowed us to determine whether a given model explains significantly different amounts of the deviance in the data (Vickers et al., 2017). All GAMM models were ranked using AIC scores and those with ΔAIC of < 2.0 from the best model were considered to have support (Burnham & Anderson, 2004). The *‘gam.check’* function was used to evaluate the adequacy of each model by examining model convergence, gradient range, Hessian matrix characteristics, and basis dimension checking results across multiple models. In general, TPC GAMM models showed a rise in the explanation of deviance when incorporating parameters that consider differences among individuals and season (Table S5).

Correlation between physiological traits and activity:

We explored the relationships between Pmax vs. minutes active, accuracy of thermoregulation (db) vs. minutes active, and efficiency of thermoregulation (E index) vs. minutes active by conducting correlation analyses. The results showed no significant relationships between either Pmax and minutes active (r = -0.09, p = 0.665), db and minutes active (r = -0.23, p = 0.256), or Eindex and minutes active (r = 0.17, p = 0.385) (Fig. S2). These findings suggest that performance traits such as Pmax, db, and E are not predictive of movement rates in our dataset.

Burnham, K. P., & Anderson, D. R. (2004). Multimodel Inference. *Sociological Methods & Research*, *33*(2), 261–304.

Kearney, M. R., & Porter, W. P. (2017). NicheMapR – an R package for biophysical modelling: the microclimate model. *Ecography*, *40*(5), 664–674.

Klinges, D. H., Duffy, J. P., Kearney, M. R., & Maclean, I. M. D. (2022). mcera5: Driving microclimate models with ERA5 global gridded climate data. *Methods in Ecology and Evolution*, *13*(7), 1402–1411.

Koenig, J., Shine, R., & Shea, G. (2001). The ecology of an Australian reptile icon: how do blue-tongued lizards (*Tiliqua scincoides*) survive in suburbia? *Wildlife Research*, *28*(3), 214–227.

Smith, K. R., Cadena, V., Endler, J. A., Porter, W. P., Kearney, M. R., & Stuart-Fox, D. (2016). Colour change on different body regions provides thermal and signalling advantages in bearded dragon lizards. *Proceedings of the Royal Society B: Biological Sciences*, *283*(1832).

Vickers, M. J., Aubret, F., & Coulon, A. (2017). Using GAMM to examine inter-individual heterogeneity in thermal performance curves for Natrix natrix indicates bet hedging strategy by mothers. *Journal of Thermal Biology*, *63*, 16–23.

**Supplementary figures & tables**


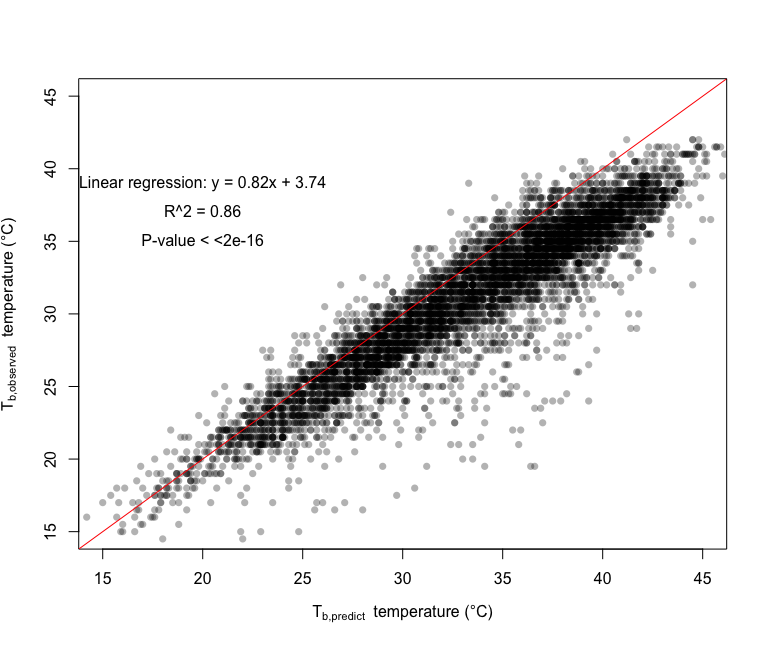


Figure S1. Comparison of predicted and core body temperatures of lizards in the field. Predicted body temperature (T_b,predict_) was estimated through laboratory adjustments of surface temperature and core body temperature measured in a laboratory thermal gradient. Field core body temperature (T_b,observed_) represents temperature recorded from implanted iButton in the field. The red line represents a perfect 1 to 1 relationship.

*
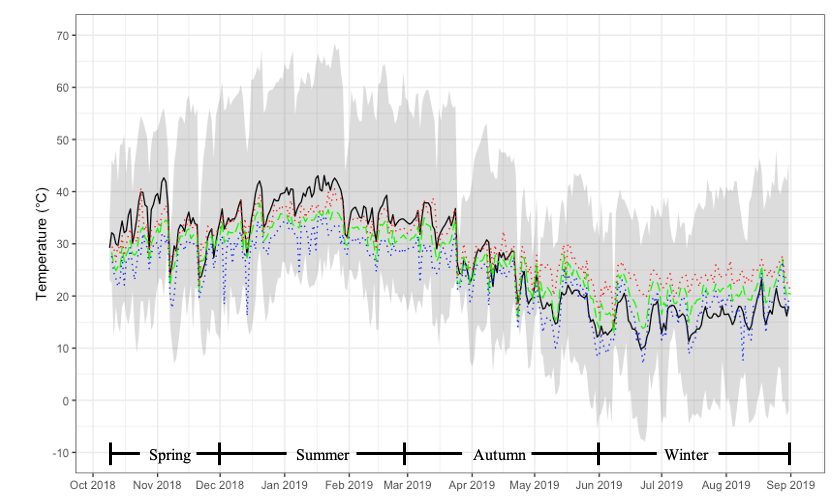
*

Figure S2. Environmental temperature range and how *Pogona vitticeps* thermoregulated during the duration of the study. Black solid lines represent the mean environmental temperatures (T_e_) for each day, and grey bands represent the daily mean minimum and maximum of T_e_. Coloured lines represent the daily mean (green), mean minimum (blue), and mean maximum (red) predicted body temperatures T_b Predict_ for a lizards during the study


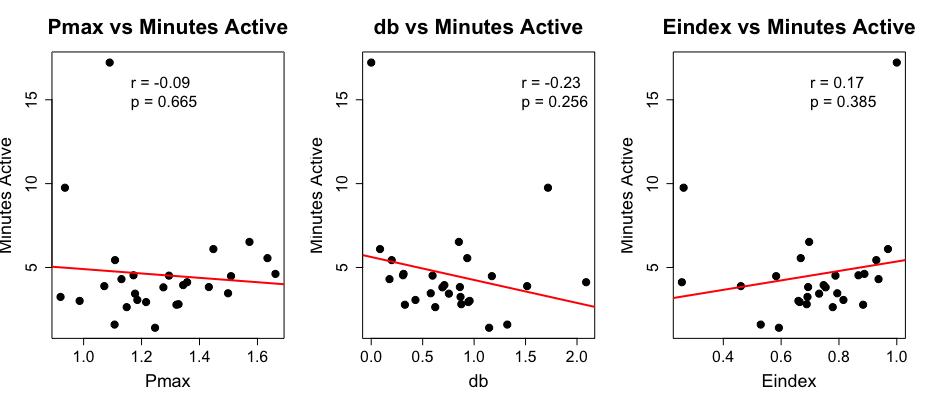


Figure S3. Relationships between maximum performance (Pmax), accuracy of thermoregulation (db), and efficiency of thermoregulation (E index) with minutes active.

Table S1. Microhabitat categories of sun exposure. At each micro-habitat category, copper pipes were placed at each cardinal direction. Sun% was calculated using a spherical densiometer.

| Exposure category | n | Definition |  |
| --- | --- | --- | --- |
| Full shade | 10 | %Sun < 25% on ground | |
| Partial shade | 10 | 25% $\geq$ % Sun $\leq$ 50% on ground | |
| Full sun | 10 | %Sun > 50% on ground | |
| Burrow | 8 | 1m within open lizard/rabbit burrow | |
| Shade-tree at 2m | 4 | Within shaded tree with %Sun > 50% | |
| Partial shade-tree at 2m | 4 | Within tree 25% $\geq$ % Sun $\leq$ 50% | |
| Open-tree at 2m | 4 | On branches of dead tree %Sun > 50% | |

Table S2. ANOVA table for predicted body temperature (T_b Predict_), accuracy of thermoregulation (d_b_), thermal quality of habitat (d_e_), and effectiveness of thermoregulation (E) for *Pogona vitticeps*. Each estimate is compared across the season, sex, and interaction. Individual lizard (or copper model ID) was treated as a repeated (random) variable. Bold values indicate significant differences.

| Model Name | Effects | Sum Sq | Mean Sq | NumDF | DenDF | F value | p value |
| --- | --- | --- | --- | --- | --- | --- | --- |
| T_b Predict_ | Sex | 9.80 | 9.80 | 1 | 37 | 0.70 | 0.41 |
|  | **Season** | **743,844.79** | **247,948.26** | **3** | **24,044** | **17,810.07** | **<0.01** |
|  | **Season x Sex** | **1,814.79** | **604.93** | **3** | **24,044** | **43.45** | **<0.01** |
| d_b_ | Sex | 5.66 | 5.66 | 1 | 33 | 0.73 | 0.4 |
|  | **Season** | **201,042.09** | **67,014.03** | **3** | **8,233** | **8,670.41** | **<0.01** |
|  | **Season x Sex** | **816.86** | **272.29** | **3** | **8,233** | **35.23** | **<0.01** |
| d_e_ | **Sex** | **14.05** | **14.05** | **1** | **304.09** | **12.65** | **< 0.01** |
|  | **Season** | **1983.80** | **661.27** | **3** | **306.74** | **595.59** | **< 0.01** |
|  | **Season x Sex** | **12.36** | **4.12** | **3** | **304.09** | **3.71** | **0.01** |
| E | Sex | 0.46 | 0.46 | 1 | 83 | 4.10 | 0.05 |
|  | **Season** | **2.14** | **0.71** | **3** | **83** | **6.34** | **<0.01** |
|  | **Season x Sex** | **1.69** | **0.56** | **3** | **83** | **4.99** | **<0.01** |

Table S3. Tukey-Kramer multiple comparisons from T_b,predict_ model (Table 2). Contrasts were extracted from the overall seasonal effect on T_b,predict_.

| contrast | estimate | SE | df | t.ratio | p value |
| --- | --- | --- | --- | --- | --- |
| **Autumn – Spring** | **-2.61** | **0.15** | **10217.31** | **-18** | **<0.01** |
| **Autumn - Summer** | **-6.83** | **0.05** | **66587.12** | **-125.52** | **<0.01** |
| **Autumn - Winter** | **5.75** | **0.05** | **67846.58** | **125.52** | **<0.01** |
| **Spring - Summer** | **-4.22** | **0.14** | **9537.75** | **-29.2** | **<0.01** |
| **Spring - Winter** | **8.37** | **0.15** | **10266.14** | **57.53** | **<0.01** |
| **Summer - Winter** | **12.58** | **0.05** | **66582.53** | **229.03** | **<0.01** |

Table S4. Tukey-Kramer multiple comparisons of overall seasonal activity rate (min/h). Activity rate was log (x+1) transformed.

| contrast | estimate | SE | df | t.ratio | p value |
| --- | --- | --- | --- | --- | --- |
| Spring - Summer | -0.28 | 0.15 | 92 | -1.86 | 0.25 |
| Spring - Autumn | 0.12 | 0.20 | 83 | 0.61 | 0.93 |
| **Spring - Winter** | **0.80** | **0.21** | **83** | **3.89** | **<0.01** |
| Summer - Autumn | 0.40 | 0.20 | 82 | 2.03 | 0.19 |
| **Summer - Winter** | **1.08** | **0.21** | **82** | **5.23** | **<0.01** |
| **Autumn - Winter** | **0.68** | **0.23** | **69** | **3.01** | **0.02** |

Table S5. General additive mixed-models for investigating how performance curves varied across season, sex and their interactions for *Pogona vitticeps*. a) accounted for all individuals in the study, b) accounted for smooth per individual, c) accounted for sex as a fixed factor, d) accounted for sex as a fixed factor and allowed for smooth per individual, e) accounted for season as a fixed factor, f) accounted for season as a fixed factor and allowed for smooth per individual, g) accounted for season and sex as a fixed factor, h) accounted for season and sex as a fixed factor and allowed for smooth per individual, i) accounted for season, sex, and the interaction as a fixed factor, and j) accounted for season, sex, and the interaction as a fixed factor and allowed for smooth per individual. Models b:j accounted for random intercept for individual lizard. Bold values indicate values were considered to have support (ΔAICc of < 2.0).

| Model  id | Model | Residual  Df | Residual  Deviance | DF | AIC | Delta  AIC | Deviance  Explained (%) |
| --- | --- | --- | --- | --- | --- | --- | --- |
| **j** | **Season + Sex + Season*Sex + s(Temperature, by = id) + (1\|id)** | **2756.35** | **262.57** | **277.61** | **1688.84** | **0** | **70.57** |
| h | Season + Sex + s(Temperature, by = id) + (1\|id) | 2760.13 | 264.88 | 273.83 | 1707.66 | 18.82 | 70.31 |
| f | Season + s(Temperature, by = id) + (1\|id) | 2760.12 | 264.93 | 273.84 | 1707.87 | 19.03 | 70.3 |
| e | Season s(Temperature) + (1\|id) | 2967.9 | 299.67 | 66.06 | 1724.75 | 35.91 | 66.41 |
| d | Sex + s(Temperature, by = id) + (1\|id) | 2766.84 | 270.68 | 267.12 | 1760.11 | 71.27 | 69.66 |
| b | s(Temperature) + (1\|id) | 2766.56 | 270.73 | 267.4 | 1760.93 | 72.09 | 69.65 |
| i | Season + Sex + Season*Sex + s(Temperature) + (1\|id) | 2989.09 | 315.19 | 44.87 | 1840.57 | 151.73 | 64.67 |
| g | Season + Sex + s(Temperature) + (1\|id) | 2992.13 | 317.28 | 41.84 | 1854.36 | 165.52 | 64.43 |
| c | Sex + s(Temperature) + (1\|id) | 2986.68 | 319.93 | 47.28 | 1888.45 | 199.61 | 64.14 |
| a | s(Temperature) | 3033.96 | 358.89 | 8.96 | 2152.09 | 463.25 | 59.77 |

Table S6. Tukey-Kramer multiple comparisons from the Pmax model that accounted for the season, sex and interaction. Contrasts were extracted from the seasonal effect.

| Contrast | Estimate | SE | df | t Ratio | p value |
| --- | --- | --- | --- | --- | --- |
| Autumn - Spring | -0.01 | 0.01 | 45 | -1.3 | 0.57 |
| **Autumn - Summer** | **0.07** | **0.01** | **45** | **13.4** | **<0.01** |
| **Autumn - Winter** | **0.10** | **0.01** | **45** | **18.6** | **<0.01** |
| **Spring - Summer** | **0.08** | **0.00** | **45** | **15.1** | **<0.01** |
| **Spring - Winter** | **0.11** | **0.01** | **45** | **18.4** | **<0.01** |
| **Summer - Winter** | **0.03** | **0.01** | **45** | **6.1** | **<0.01** |

Table S7. Tukey-Kramer multiple comparisons from the Pmax model that accounted for the season, sex and interaction. Contrasts were extracted from season and sex interaction.

| Contrast | Season | Estimate | SE | df | t Ratio | p value |
| --- | --- | --- | --- | --- | --- | --- |
| Female - Male | Autumn | 0.03 | 0.1 | 39 | 0.34 | 0.74 |
| Female - Male | Spring | -0.13 | 0.1 | 38 | -1.33 | 0.19 |
| Female - Male | Summer | -0.15 | 0.1 | 38 | -1.49 | 0.14 |
| Female - Male | Winter | 0.01 | 0.1 | 39 | 0.14 | 0.89 |

Table S8. Model comparisons of spring survival probability (φ) for *Pogona vitticeps*, depending on sex, movement (min/h), accuracy of thermoregulation (d_b_), effectiveness of thermoregulation (E), and maximum performance (P_max_). Sex interactions for d_b_ and E were accounted for because of the differences between males and females during the spring (Table S2). Values within the brackets are nested variables, and variables outside of brackets are covariates. Bold values indicate values were considered to have support (ΔAICc of < 2.0).

| Model | AICc | Δ AICc | AICc Weights | Model Likelihood | Number of Parameters | Deviance |
| --- | --- | --- | --- | --- | --- | --- |
| **φ(Sex)P_max_** | 27.67 | **0.00** | **0.79** | **1.00** | **3** | **20.63** |
| φ(.)P_max_ | 31.47 | 3.80 | 0.12 | 0.15 | 2 | 26.97 |
| φ(.) | 34.99 | 7.30 | 0.02 | 0.03 | 1 | 32.82 |
| φ(Sex) | 35.31 | 7.64 | 0.02 | 0.01 | 2 | 30.81 |
| φ(db) | 36.52 | 8.84 | 0.01 | 0.01 | 2 | 32.02 |
| φ(.)E | 36.57 | 8.90 | 0.01 | 0.01 | 2 | 32.07 |
| φ(.)T_opt_ | 36.86 | 9.18 | 0.01 | 0.01 | 2 | 32.36 |
| φ(Sex)d_b_ | 37.02 | 9.35 | 0.01 | 0.01 | 3 | 29.98 |
| φ(.)Activity | 37.31 | 9.64 | 0.01 | 0.01 | 2 | 32.81 |
| φ(Sex)T_opt_ | 37.35 | 9.68 | 0.01 | 0.01 | 3 | 30.31 |
| φ(Sex)E | 37.36 | 9.68 | 0.01 | 0.01 | 3 | 30.31 |
| φ(Sex)Activity | 37.77 | 10.10 | 0.01 | 0.01 | 3 | 30.73 |
